# Ultra−Wide Range Vibration Frequency Detection Sensors Based on Elastic Steel Triboelectric Nanogenerators for Intelligent Machinery Monitoring

**DOI:** 10.3390/nano12162790

**Published:** 2022-08-14

**Authors:** Xili Huang, Cheng Zhang, Hongchen Pang, Zhiqiang Zhao, Qianxi Zhang, Xiaoning Li, Xianzhang Wang, Fang Lin, Bo Li, Xinxiang Pan

**Affiliations:** 1 School of Electronic and Information Engineering, Guangdong Ocean University, Zhanjiang 524088, China; 2 School of Mechanical and Power Engineering, Guangdong Ocean University, Zhanjiang 524088, China

**Keywords:** condition monitoring, vibration sensor, high-frequency, triboelectric-nanogenerator

## Abstract

Vibration measurement and analysis play an important role in diagnosing mechanical faults, but existing vibration sensors are limited by issues such as dependence on external power sources and high costs. To overcome these challenges, the use of triboelectric nanogenerator (TENG)−based vibration sensors has recently attracted attention. These vibration sensors measure a small range of vibration frequencies and are not suitable for measuring high-frequency vibrations. Herein, a self-powered vibration sensor based on an elastic steel triboelectric nanogenerator (ES−TENG) is proposed. By optimizing the elastic steel sheet structure and combining time-frequency transformation and filtering processing methods, the measurement of medium- and high-frequency vibrations is achieved. These results demonstrate that the ES−TENG can perform vibration measurements in the range of 2–10,000 Hz, with a small average error (~0.42%) between the measured frequency and external vibration frequency values. Therefore, the ES−TENG can be used as a self-powered, highly-accurate vibration sensor for intelligent machinery monitoring.

## 1. Introduction

Components in machinery and equipment often fail owing to material deterioration or the lack of predictive maintenance, which degrades the mechanical movement and leads to potential safety issues [1]. The real-time monitoring of operating conditions is a necessary preventive measure, in addition to appropriate working conditions, meticulous construction specifications, and regular maintenance, to maintain machinery in good operating condition [2]. Vibration measurement and analysis play an important role in diagnosing mechanical faults. Fault diagnosis and condition monitoring can prevent production accidents by processing and analyzing the vibration signals of machinery and equipment and by comparing the signal changes over different periods. However, existing research has mainly focused on the analysis of vibration signals, and studies on vibration sensors are limited [3,4]. Traditional vibration monitoring sensors are powered by wires or batteries. The increase in the level of complexity of sensor arrays leads to an increase in the number of sensors and the expansion of the range of distribution. Consequently, conventional vibration sensors are less suited for the current sophisticated applications. They are not capable of continuous, real-time, and accurate monitoring of the working conditions. Therefore, finding a self-powered, highly accurate, and robust vibration sensor for intelligent machinery monitoring is of crucial importance.

The triboelectric effect, the transfer of charge associated with rubbing or contacting two materials, has been known for at least twenty-five centuries [5]. However, the fundamental mechanism of charge transfer is still not understood. LD Marks et al. proposed a model where flexoelectric band bending due to local asperity contacts drives triboelectric charge transfer in non-metals. This work indicates a general ratcheting mechanism for triboelectric transfer and strong experimental evidence that flexoelectric band bending is of fundamental importance for triboelectric contacts [6]. The triboelectric nanogenerator (TENG) was first proposed by Wang et al. [7] in 2012 as a new means of generating mechanical energy based on Maxwell’s displacement current principle, which combines triboelectricity and electrostatic induction to efficiently convert mechanical energy from the environment into electrical energy [8,9,10]. TENGs have the advantages of light weight, diverse raw materials, a simple structure, and low cost, and they can be applied to different fields of energy harvesting [11,12,13]. Significant advancements have been achieved in the field of vibrational energy harvesting. For instance, Wang et al. designed a spring-assisted hybrid TENG that can generate approximately 57.6 mW peak power at 2 Hz [14]. Zhang et al. proposed a new paper-based rhombic TENG, a novel design that helps the device achieve higher results [15]. Xu et al. fabricated a soft and robust spring-loaded TENG for harvesting vibration energy in desired directions, achieving a power density of 45 mW/m^2^ under certain conditions [16].

Various self-powered sensors based on TENGs have been developed, including vibration [17], pressure [18], displacement [19], angle [20], haptic [21], and wind speed sensors [22]. Wu et al. developed a new spherical TENG with a measurement range of 0–8 Hz and a testing error of less than 2% for downhole drilling vibration, and it can reach a maximum output voltage of 70 V, a maximum current of 3.3 × 10^−5^ A, and a maximum power of 10.9 × 10^−9^ W at 8 Hz when a 10-ohm resistor is connected [23]. Du et al. proposed a bouncing ball triboelectric nanogenerator (BB-TENG) based vibration sensor with a high signal-to-noise ratio of 34.5 dB and an average error of less than 0.05% for vibration frequencies from 10 Hz to 50 Hz [24]. Wang et al. demonstrated a vertical contact-separation based freestanding triboelectric nanogenerator (CF-TENG). This device shows the capability of measuring amplitude up to 16 mm [25]. However, these vibration sensors are not suitable for measuring medium- to high-frequency vibrations owing to their small measurement frequency range. To meet the requirements of industrial machine condition monitoring (typically 10–1000 Hz), Idiris Mehamud et al. designed a two spring-assisted TENG with a wider frequency detection range (0–1200 Hz), which was realized through structural design supported by computed natural frequency simulation [26]. However, previous studies on TENG-based vibration sensors have been limited to the low and medium-frequency range, which are not suitable as sensors for measuring high-frequency vibrations (more than 1000 Hz). The conditions and performance of the TENG-based vibration sensors mentioned are summarized in Table 1.

Therefore, the elastic steel TENG (ES−TENG) sensor has been designed as a novel self-powered, highly accurate, and robust vibration sensor. In this study, a hollow structural solution is proposed to reduce the stiffness of the spring structure and enable the ES−TENG to achieve higher frequency and small amplitude vibration detection. Time-frequency conversion and filtering are combined by optimizing the structure of the elastic steel sheet to facilitate the medium- and high-frequency measurements. The results show that the ES−TENG can perform vibration measurements in the range of 2–10,000 Hz. 

## 2. Materials and Methods

### 2.1. Working Principle and Theoretical Model of the ES−TENG Sensor

**Figure 1:**
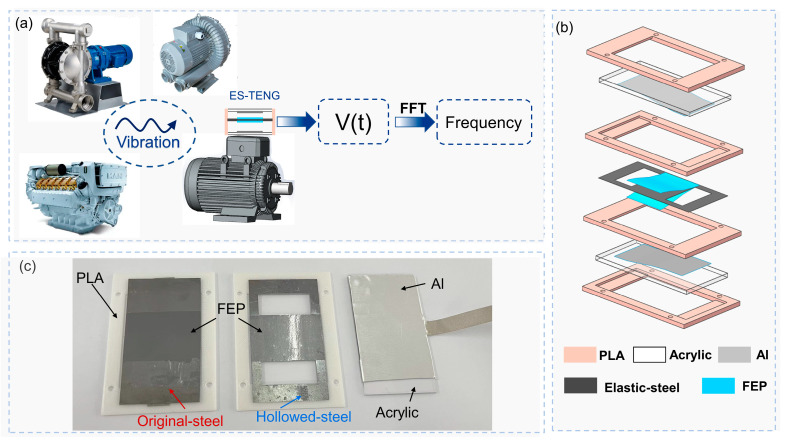
Application scenario and specific structure of the elastic steel triboelectric nanogenerator (ES−TENG) sensor. (**a**) Application scenario of ES−TENG; (**b**) explosive view of ES−TENG; (**c**) photograph of ES−TENG.

This paper presents a highly accurate vibration sensor based on the TENG in the free-standing mode, with the application scenario and specific structure of ES−TENG, shown in Figure 1. The sensor has two fixed pole plates made of acrylic on the top and bottom, with aluminum films of 0.1 mm thickness attached to the inner side as the electrodes of the sensor, which was sealed by four poly lactic (PLA) housings. In the middle, an elastic steel structure, with 0.03 mm-thick fluorinated ethylene propylene (FEP) membranes embedded on the upper and lower surfaces, was present. Details of the structure and fabrication of this device are provided in Section 2.2. 

**Figure 2:**
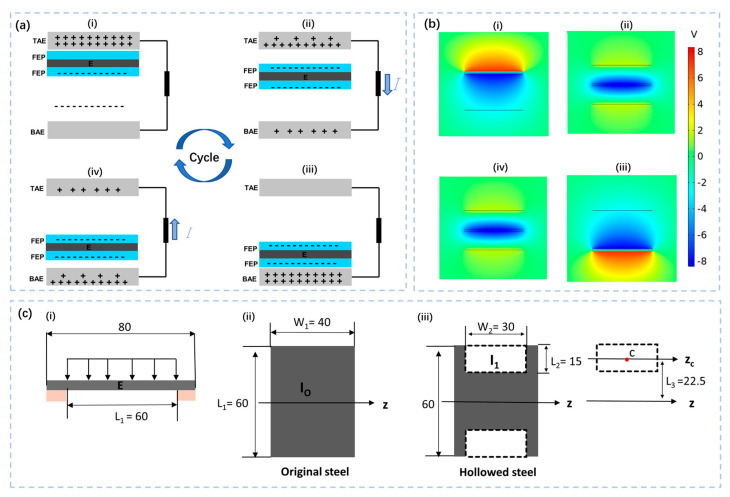
Working principle and simulation of the ES−TENG sensor. (**a**) Working principle of the ES−TENG; (**b**) simulation graphic of COMSOL Multiphysics; (**c**) force analysis diagram of elastic steel.

The working principle of the sensor is explained using the unidirectional motion of the FEP film on the elastic steel sheet. The elastic steel sheet is fixed in the middle of the device, and no charge transfer occurs if there is no external excitation. When the external vibration excitation is provided, the elastic steel sheet deforms, and the FEP film on the elastic steel sheet will contact the aluminum electrode, and electron transfer occurs. The movement of the negatively charged FEP film up and down between the top aluminum electrode (TAE) and the bottom aluminum electrode (BAE) causes the electrons to move between the two electrodes through an external circuit to generate an electrical signal, as shown in Figure 2b(i). The specific working process is as follows: when the FEP film is in contact with the TAE (Figure 2a(i), several electrons from the TAE are transferred to the FEP film. Afterward, when the FEP film moves from the TAE toward the BAE (Figure 2a(ii)), because the TAE has a higher potential than the BAE, electrons will move from the BAE to the TAE through the external circuit, thereby generating a positive transient current. When the FEP film reaches the BAE (Figure 2a(iii)), several electrons in the BAE are transferred to the FEP film. Subsequently, when the FEP film moves from the BAE to the TAE (Figure 2a(iv)), the higher potential of the BAE than TAE drives the electrons to move from the TAE to the BAE through the external circuit, thereby generating a reverse transient current. Thereafter, the FEP film regains the position shown in Figure 2a(i), completing a full operating cycle. The FEP is an electret material in which the frictional charge on the surface persists for a long time. The movement of the FEP film between the two electrodes will cause the potential difference owing to electrostatic induction, even if no physical contact exists between the FEP and aluminum electrodes in the later working stages, and an electrical signal will be generated. Therefore, the as-proposed ES−TENG can accurately detect external vibration in both contact and non-contact modes.

To confirm the above analysis, the potential distribution of the electrostatic field when the charged FEP film of the ES−TENG is located at different positions under open-circuit conditions is simulated using the COMSOL Multiphysics software, as shown in Figure 2b. With sufficient external excitation, the FEP membrane on the elastic steel sheet moves continuously back and forth between the two electrode plates, and the induced potential difference between the two electrode plates will drive the electrons to flow periodically in the external circuit and generate an electrical signal related to the vibration state of the membrane. The vibration state of the membrane is related to the state of external excitation. Therefore, analyzing the electrical signal of the sensor can determine the external vibration state.

The theory of freestanding triboelectric-layer-based nanogenerators suggests that the total negative charge on both FEP membranes is, ideally, equal to the amount of positive charge on both electrodes. The controlling equation of ES−TENG can be written as [25,27]:

(1) $$V=-\frac{1}{C}Q+V_{OC}=-\frac{d_{0}+g}{\varepsilon_{0}s}Q+\frac{2\sigma x}{\varepsilon_{0}}$$

where *C*, *Q*, *V_OC_*, *d*_0_, *g*, *ε*_0_, *S*, *σ*, and *x* are the capacitance of the ES−TENG unit, transferred charge, open-circuit voltage, effective dielectric medium thickness, total air-gap thickness between two electrodes, effective contact area of the copper electrode layer, dielectric constant in vacuum, charge density on the FEP surface, and separation distance between the electrode layer and FEP surface, respectively.

Under the minimum achievable charge reference state (MACRS), the short circuit transferred charges (*Q*) and open-circuit voltage (*V_OC_*) can be calculated as follows:

(2) $$Q=\frac{2\sigma Sx}{d_{0}+g}$$

(3) $$V_{OC}=\frac{2\sigma x}{\varepsilon_{0}}$$

In fact, *x* is also the disturbance displacement of the spring steel sheet. Therefore, the displacement of the spring steel is a crucial factor that determines the electrical output of ES−TENG. As shown in Figure 2c(i), the elastic steel can be simplified as a simply-supported beam. The deformation of the elastic steel sheet is mainly bending when the external load is uniformly distributed on the beam. The deflection displacement of the spring steel can be calculated by the following formula:

(4) $$x=-\frac{mX^{2}}{2K}$$

(5) K=EI

where *x* is the deflection displacement of the cantilever beam and *X* is the distance from the origin to the constraint segment. *K* is the stiffness, *E* is elastic modulus, and *I* is the moment of inertia of the section.

For a certain material, whose elastic modulus is determined, the stiffness can be changed by varying the moment of inertia of the section. For the original steel (Figure 2c(ii)), the moment of inertia of the section can be expressed as:

(6) $$I_{O}=\frac{W_{1}L_{1}{}^{3}}{12}$$

For the hollowed steel (Figure 2c(iii)), the moment of inertia of the section can be written as:

(7) $$I_{H}=I_{O}-2I_{1}=\frac{W_{1}L_{1}{}^{3}}{12}-\frac{W_{2}L_{2}{}^{3}}{6}-W_{2}L_{2}L_{3}{}^{2}$$

From the above equations, it can be found that the stiffness *K* and the moment of inertia of the section *I* of the hollowed steel are effectively reduced, which allows the deflection displacement *x* to be increased at the same external vibration conditions. Therefore, the output performance of ES−TENG with a hollow structure will be significantly improved compared with that of the original structure.

### 2.2. Fabrication of the ES−TENG Sensor

The sample preparation was divided into two steps. The first step was material selection. Considering the quantification of the frictional electron sequences of commonly used materials, properties of ES−TENG, cost, and availability of materials, FEP and aluminum foil were selected as the frictional materials for ES−TENG owing to their large differences in ionization energy and electron affinity [28]. Aluminum foil was also used as the electrode material for the ES−TENG sensor. A 65 Mn hardened manganese steel sheet was selected for the elastic steel sheet, which has excellent elastic deformation capability and can withstand a significant amount of load without being permanently deformed. The size of the elastic steel sheet was 80 × 40 × 0.1 mm^3^, and the hollowed structure was designed (the hollowed structure was produced via laser cutting). The support plate was produced from an acrylic plate, and the sealing structure was three-dimensionally (3D) printed from PLA.

The second step was sample assembly. Two pieces of the aluminum foil with dimensions of 40 × 30 × 0.1 mm^3^ were attached to the inner walls of two acrylic plates (80 × 30 × 1 mm^3^) as positive frictional electric layers. Two pieces of the FEP film with dimensions of 40 × 30 × 0.08 mm^3^ were attached to the two surfaces of the elastic steel sheet as the negative friction layers. The PLA seal structure was used to fix the two acrylic plates and elastic steel sheet and was modeled and designed using the SolidWorks software. The designed PLA seal structure model was then imported to a 3D printer for printing and molding. After connecting the aluminum foil on the two acrylic panels to the external circuit, the parts were fixed using the sealing structure.

### 2.3. Experimental Setup

**Figure 3:**
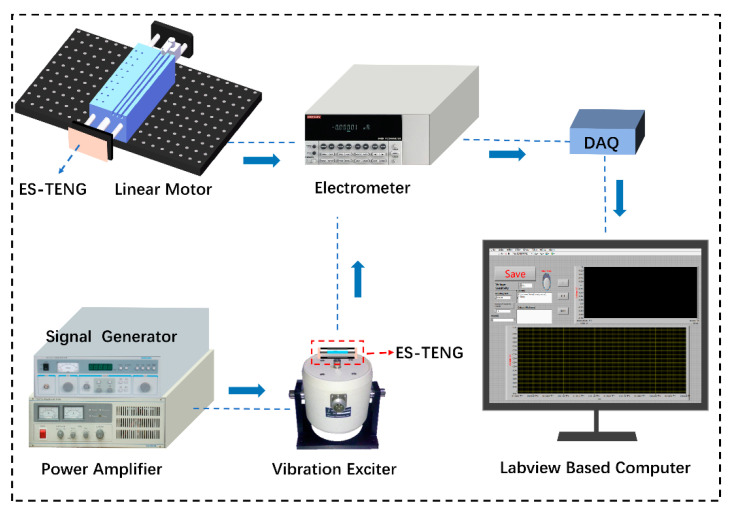
Schematic of the experimental apparatus for assessing ES−TENG sensor performance.

Figure 3 shows the experimental system for the ES−TENG performance test. The test system consists of a linear motor, a signal generator, a power amplifier, an exciter, an electrostatic meter, data acquisition equipment, and the LabVIEW software. The signal produced by the signal generator is amplified by the power amplifier and used to drive the shaker’s movable plate to generate vibration excitation. The signal generator can be used to generate different types of vibration signals, such as sine and triangular signals. The equation of motion and the maximum acceleration of the vibration exciter, which can be estimated using the calculus theory, can be expressed as:

(8) $$a=A{\left(2\pi f\right)}^{2}$$

where *A* is the vibration amplitude, *a* is the maximum acceleration of the vibration exciter, and *f* is the vibration frequency of the vibration exciter.

The vibration frequency can be continuously adjusted using the signal generator from 2 Hz to 10 K Hz, and the amplitude depends on the output voltage level of the power amplifier. The ES−TENG transducer is firmly attached to the shaker’s moving plate. The elastic steel sheet in the ES−TENG sensor will deform as the moving plate vibrates, changing the position of the FEP membrane between the TAE and BAE, thereby generating an electrical signal. The wire at the rear of the aluminum electrode is connected to an electrostatic meter, and the electrical signal is collected by DAQ and then transmitted to a LabVIEW-based computer. A vibrometer (VM188A, Sanliang, Dongguan, China) was used to measure the maximum acceleration of the shaker to determine the shaker’s amplitude and to verify the performance of the ES−TENG sensor.

Owing to the performance limitations of the shaker equipment, experiments at low frequencies and high amplitudes could not be performed. To further investigate the performance of the ES−TENG comprehensively, a linear motor was added to the experimental system to test the output characteristics at low frequencies (1–10 Hz) and different amplitudes (1–5 mm).

## 3. Results and Discussion

### 3.1. Output Performance Characteristics of the ES−TENG Sensor

**Figure 4:**
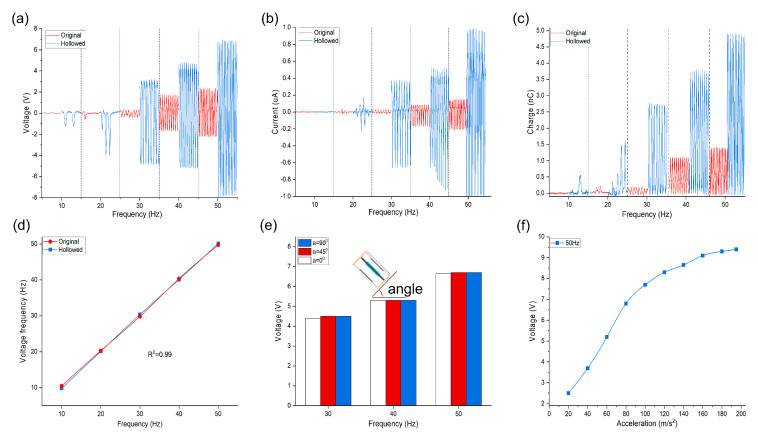
Output performance of ES−TENG sensors (hollow and original) at different frequencies with a fixed amplitude of 2 mm: (**a**) *V_OC_*, (**b**) short-circuit current, and (**c**) transferred charge. (**d**) FFT results of the applied vibration frequency and *V_OC_* signals; (**e**) *V_OC_* values of ES−TENG at different vibration directions; (**f**) voltage variation curves for different accelerations at 50 Hz.

The ES−TENG was tested using the shaker over a range of 10 to 50 Hz at a fixed amplitude (2 mm), and the findings were compared to results from an original non-hollow structure, Figure 4. The *V_OC_*, *I_SC_* and *Q* of both the hollowed structure and original piece are approximately zero or unstable when excited with an external vibration frequency of 10 Hz. In the range of 20 to 50 Hz, the amplitude of the output electrical signal increases with increasing frequency. It can be explained that under the same amplitude, the higher the vibration frequency, the greater the acceleration of the shaker acting on the FEP and the greater the impact of the FEP in contact with the aluminum electrode, and then the more frictional charges generated. For the hollowed structure, a significant increase in *V_OC_*, *I_SC_* and *Q* was observed: *V_OC_* increased from 3.5 V at 20 Hz to 15 V at 50 Hz, *I_SC_* from 0.2 μA at 20 Hz to 0.9 μA at 50 Hz, and *Q* from 1.5 nC at 20 Hz to 4.8 nC at 50 Hz. For the original structure: *V_OC_* increased from 0.5 V at 20 Hz to 4.5 V at 50 Hz, *I_SC_* from 0.04 μA at 20 Hz to 0.21 μA at 50 Hz, and *Q* from 0.3 nC at 20 Hz to 1.4 nC at 50 Hz. The original non-hollow test piece shows a smaller but steady trend of increase. We suggest that the hollowed structure reduces the structural stiffness, allowing for larger and more stable deformation of the steel. The *V_OC_* is approximately 1.1 V when the vibration frequency of the shaker is 10 Hz, Figure 4a. The FFT processing of *V_OC_*, measured vibration frequency is 9.83 Hz (the relative error is 1.7%), indicating a very high measurement accuracy. Figure 4d shows that in the range of 10 to 50 Hz, the frequency of the voltage signal after FFT processing has a good linear relationship with the vibration frequency of the shaker, with a correlation coefficient *R*^2^ of 0.99, demonstrating excellent vibration main frequency monitoring characteristics. These findings illustrate that the voltage signal of ES−TENG can be used to obtain the real-time vibration main frequency of mechanical equipment via fast Fourier transform analysis to monitor the working conditions of the machine.

The aim is now to study the output characteristics of ES−TENG under different vibration direction conditions with a fixed output amplitude of the modal shaker (A = 0.5 mm) and different vibration direction angles (0°, 45°, and 90°) over a range of vibration frequencies (30 Hz, 40 Hz, and 50 Hz). Figure 4e shows that the *V_OC_* gradually increases as the vibration frequency increases. The output values of the ES−TENG are approximately the same regardless of the value of the vibration angle, proving that it can harvest the vibration energy in different directions. The output characteristics of the ES−TENG are significantly affected by different acceleration conditions, as shown in Figure 4f. The *V_OC_* increases significantly from 2.5 to 8.2 V with increasing acceleration in the interval of 20–120 m/s^2^ at a constant frequency and more gradually in the interval of 140–190 m/s^2^. This can be interpreted as indicating that, under the premise of constant frequency, the acceleration increases and the FEP impact force also increases; thus, the frictional contact effect between FEP and aluminum electrode gradually increases and finally reaches the best effect, so the output performance will increase and then tends to be stable. Moreover, the FEP moves with a small stroke, and after the amplitude becomes too large, the speed that the FEP can reach is limited, so the impact force does not increase indefinitely. For a determined device, the frictional charge generated will reach a limiting range of values regardless of the external conditions applied, and there will always be a range of the best output performance. According to the above, the rational design of the electrode plate interrogation distance of ES−TENG can effectively improve the power generation performance of this device according to the different vibration environments, which will be discussed in our future work. For example, a larger electrode spacing device should be selected with a larger amplitude environment, and, accordingly, a smaller electrode spacing device should be selected with a smaller amplitude environment.

### 3.2. Working Range of Vibration Frequency Sensing

**Figure 5:**
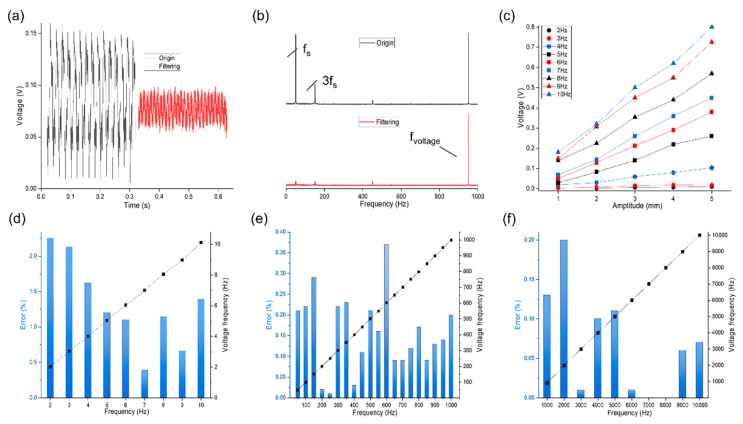
Working range of the ES−TENG sensor. (**a**) Comparison between the original and filtered voltage signals of ES−TENG at 950 Hz; (**b**) corresponding spectrograms; (**c**) voltage values of ES−TENG with different amplitudes at 2–10 Hz. FFT plots of *V_OC_* signals: (**d**) 2–10 Hz; (**e**) 10–1000 Hz; (**f**) 1000–10,000 Hz.

The electrical signals of the ES−TENG are unstable and weak at low vibration frequencies or low amplitudes of the shaker, but the as-developed ES−TENG outperformed the original all-solid structure. Therefore, subsequent experiments were conducted with the hollow ES−TENG device in the vertical vibration direction. Experiments were conducted to investigate the performance of the ES−TENG in the measurement of high-frequency vibration in the range of 100–1000 Hz and 1000–10,000 Hz at intervals of 50 Hz and 1000 Hz, respectively. In all experimental results, an interference signal of 150 Hz was observed in the vibration spectrogram, in addition to that of 50 Hz. We determined that 50 and 150 Hz are the power supply frequency f_s_ and the third harmonic 3f_s_, respectively. The oscillator operates with a low amplitude (especially at high frequencies, wherein the vibration amplitude is at the micron level), and the presence of the f_s_ and 3f_s_ will affect the accuracy of ES−TENG. Therefore, we constructed a digital band-stop filter with a bandwidth of 1 Hz using a Python program to remove the frequency components of 50 and 150 Hz. Representative results of the experiment after implementing the filtering process and FFT are shown in Figure 5a,b. This signal processing allows the maximum monitoring frequency of the ES−TENG to be increased to 10,000 Hz. The performance of the ES−TENG in measuring low-frequency vibration with variable amplitudes is shown in Figure 5c, indicating that larger amplitudes result in larger *V_OC_* values at the same frequency; at 2 Hz, an amplitude of 2 mm is needed for detection. Figure 5d–f shows that the ES−TENG can detect vibration in the range of 2 to 10,000 Hz, and the frequency of the voltage signal after FFT processing is in good agreement with the vibration frequency of the shaker, with an average error of approximately 0.42%.

### 3.3. Practical Applications of the ES−TENG Sensor

**Figure 6:**
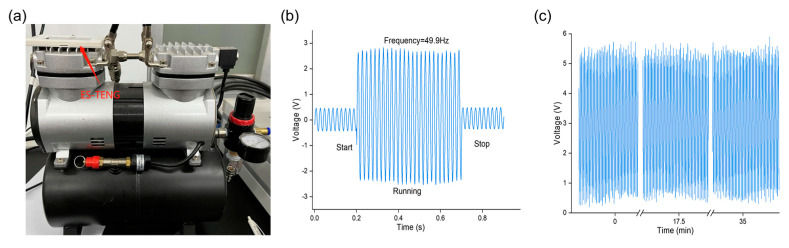
Practical application of ES−TENG. (**a**) ES−TENG installed on the air compressor; (**b**) monitoring of the working condition of the air compressor; (**c**) endurance test results.

The ES−TENG sensor can monitor the start, run, and stop status of an air compressor, as shown in Figure 6a,b, which demonstrates its capability as a precise vibration sensor. The two-piston, two-cylinder compressor operates at 1500 rpm, with both cylinders alternating in one cycle; therefore, its operating frequency is 1500 × 2/60 = 50 Hz. The vibration frequency detected by the ES−TENG sensor is 49.99 Hz, which is in good agreement with the actual vibration frequency of the compressor. This indicates that the ES−TENG vibration sensor exhibits good performance in monitoring mechanical vibration frequencies. Figure 6c shows that the ES−TENG sensor retains this performance for detecting frequencies with excellent accuracy after 35 min of continuous operation at 50 Hz. The output signal of ES−TENG maintains good stability after more than 100,000 operating cycles, showing its significant potential for applications in vibration frequency monitoring.

## 4. Conclusions

In this study, an ultra-wide-range vibration frequency detection sensor based on elastic steel triboelectric nanogenerators for intelligent machinery monitoring was fabricated and tested. Both the accuracy and detection range of the vibration sensor are significantly improved due to the reduction in the stiffness of the elastic steel sheet owing to the hollow structure. The vibration sensor displayed a high sensitivity and strong stability. The experimental results show that the relative error in the frequency value measured by ES−TENG is small (approximately 0.42%) when compared with the external vibration frequency value. Compared with the vibration sensors commonly used for mechanical condition monitoring, the ES−TENG has the following advantages:

1. Self-powered characteristics: The ES−TENG is based on the TENG with the free-standing mode to harvest vibration energy and can continuously output signals without power supply.
2. Simple structure and good applicability: The ES−TENG has a simple sandwich structure with good stability. The output performance of the ES−TENG is independent of the placement angle and can be used to detect vibration in any direction.
3. High reliability: The ES−TENG has contact and non-contact working modes. In the non-contact mode, the wear of dielectric materials can be effectively reduced; therefore, the ES−TENG has good operational reliability.
4. Low cost: Compared to traditional vibration sensors, ES−TENG is substantially more affordable to produce. This will significantly reduce the fabrication cost of vibration sensors.

Considering the aforementioned advantages, the as-developed ES−TENG vibration sensor demonstrates significant potential for application.

## Figures and Tables

**Table 1:** The conditions and performance of different TENG-based vibration sensors.

Ref.	Frequency Range/Hz	Amplitude Range/mm	Maximum Voltage/V	Maximum Current/μA	Maximum Charges/nC	Maximum Power Density
[23]	1–8	-	70	33	2.9	-
[24]	10–50	1–8	32.5	1.6	3.5	3 W/m^2^
[25]	1–15	0–16	165	2.0	-	17 mW/m^2^
[26]	0–1200	-	200	0.9	-	-
This Work	2–10,000	0–5	15	0.9	4.8	-

## Data Availability

Not applicable.
